# Supplementary material for: Homing gene drives can transfer rapidly between Anopheles gambiae strains with minimal carryover of flanking sequences
Source: Nat Commun. 2024 Aug 10;15:6846. doi: 10.1038/s41467-024-51225-9 (PMC11315913; doi:10.1038/s41467-024-51225-9)
Supplement: Supplementary file 1 — Supplementary Information [file 41467_2024_51225_MOESM1_ESM.pdf]

Supplementary material: Homing gene drives can transfer rapidly between *Anopheles gambiae* strains with minimal carryover of flanking sequences

Supplementary Note 1 – Sequencing Controls

To control for any errors produced by PCR of pooled samples (such as hybridisation of amplicons to form chimeras, erroneous base changes, etc.), or produced by Cas9 activity at the target site, control samples were sequenced. An *en masse* cross of 20 *vas2*-5958 males and 20 N'Gousso females was performed to produce hybrid offspring, which were reared to adulthood. Six hybrid female adults were chosen for sequencing of the region left and right of the *zpg*-7280 cut site, which was not targeted by Cas9 in this colony and was therefore assumed to be free of the effects of Cas9 activity. As a further precaution the samples were dissected to remove all germline tissue to prevent sequencing of tissue that may have been cut by the germline-expressed Cas9. DNA was extracted from each individually as per the methods in the main paper. A pool was also created from all six DNA extractions by combining an equal volume of each.

For each sample, including the pool, ~350 bp regions left and right of the *zpg*-7280 cut site were amplified using the primers in Table S2 and methods in the main text. Samples were sent for Illumina MiSeq sequencing and reads processed using CRISPResso2 in the same manner as samples in the main text (Accession: PRJNA1043640). The percentage abundance of all alleles >0.5% was visualised (**Figure S1**); the most abundant two reads in the individually sequenced samples segregated well from the rest of the reads and were therefore taken to be the two true haplotypes present in each hybrid individual. These true haplotypes were identified in the pools (**Figure S1**), allowing the development of a strategy to interpret pooled samples and distinguish true haplotypes from erroneous ones.

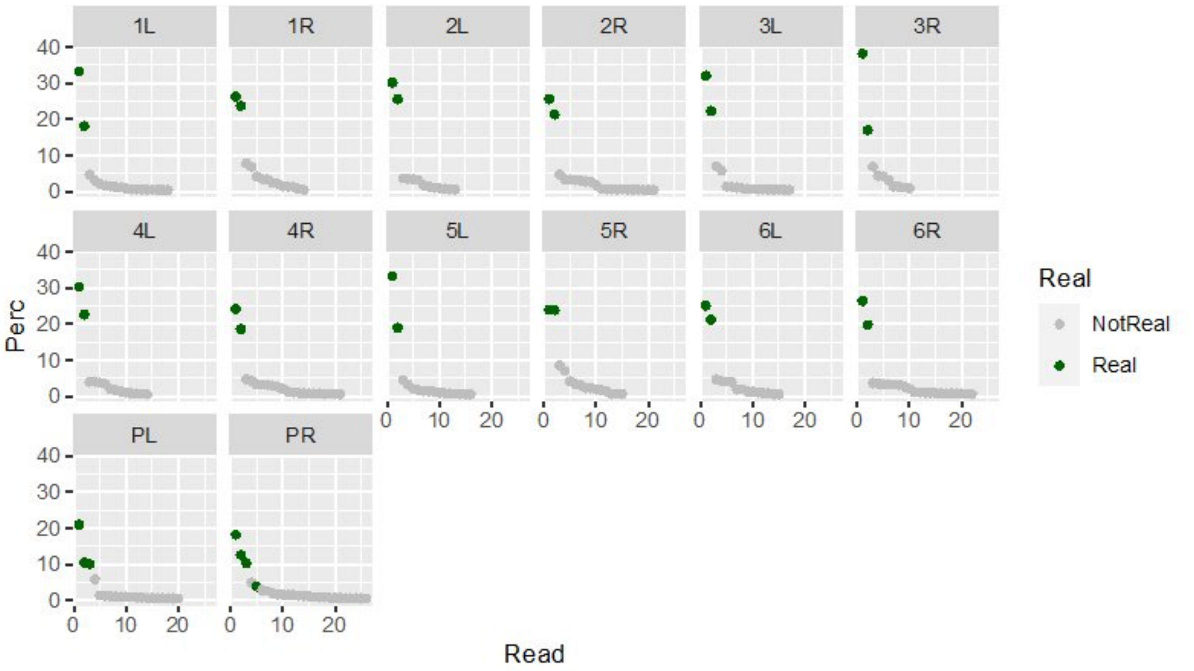

Supplementary Figure 1 - Relative percentage abundance of unfiltered alleles at >0.5% left and right of the cut site in control samples 1-6 and the pooled sample P. The most common two alleles in individual samples are identified as 'Real' alleles in samples 1-6, which are then identified in the pooled (P) samples.

In the six control samples there were three unique haplotypes on the left of the cut site and five on the right. All three haplotypes on the left were identified as the three most abundant alleles in the pooled sample; however, on the right of the cut site only four out of five haplotypes were identified (**Figure S2 and S3**). The fourth most abundant allele on the right of the cut site did not match any of the haplotypes identified in the individual samples, with 2-7 mismatches (0.5-1.9% difference).

The proportion of reads matching each of the true haplotypes varied within samples both from individuals and from pools, demonstrating the stochasticity of sequencing results despite the assumed equal representation of each haplotype in the original sample. There was a characteristic pattern observed in the erroneous alleles in each individual sample: alleles identified as not real almost always matched expected chimeras of the two true haplotypes (made up partially of both reads, in mosaic format), and rarely contained sequences not found in either of the two true haplotypes (**Figure S4**). This further demonstrates the importance of using controls to determine true haplotypes in this study; expected homed haplotypes with carryover of genetic material adjacent to the gene drive would appear similar to PCR chimeras.

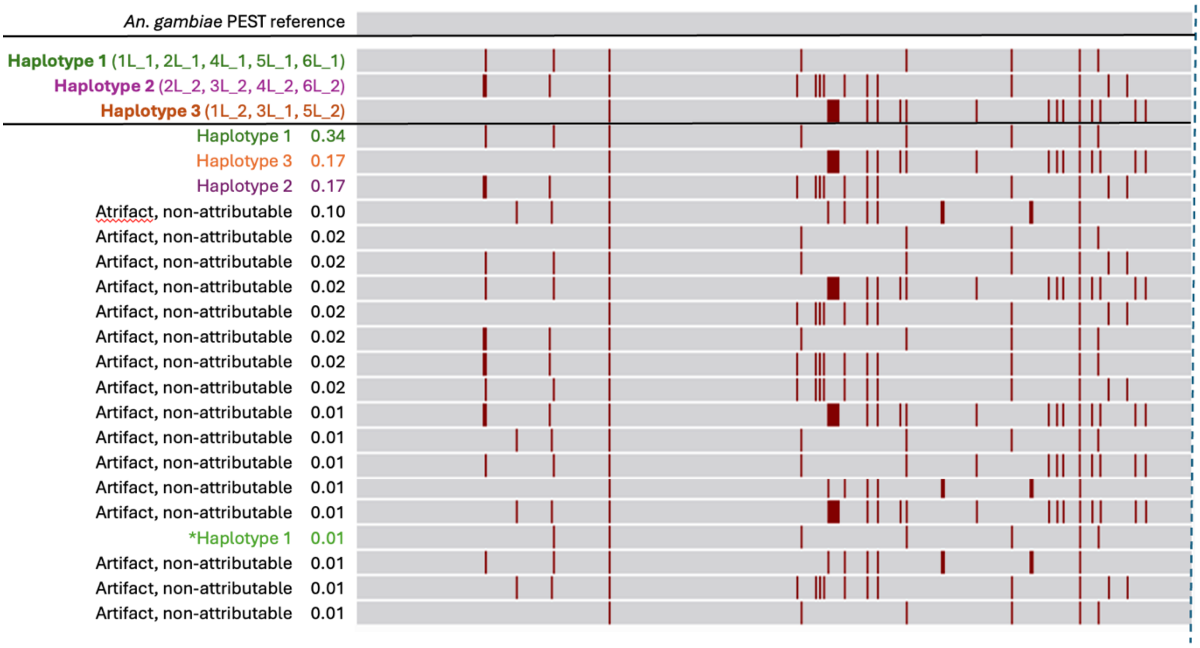

Supplementary Figure 2 – All alleles present at ≥0.5% of reads in pooled control samples on the left of the *zpg*-7280 cut site. Alleles were aligned against the *Anopheles gambiae* PEST reference sequence, with the three expected haplotypes based on the sequences from individual samples shown underneath the PEST reference. The most common alleles match the three expected haplotypes. The remaining alleles are artifacts which have more than one mismatching base compared to the expected haplotypes, except for one allele which was 1 bp dissimilar to Haplotype 1 and can therefore be reasonably assumed to originate from that haplotype (marked with an asterisk).

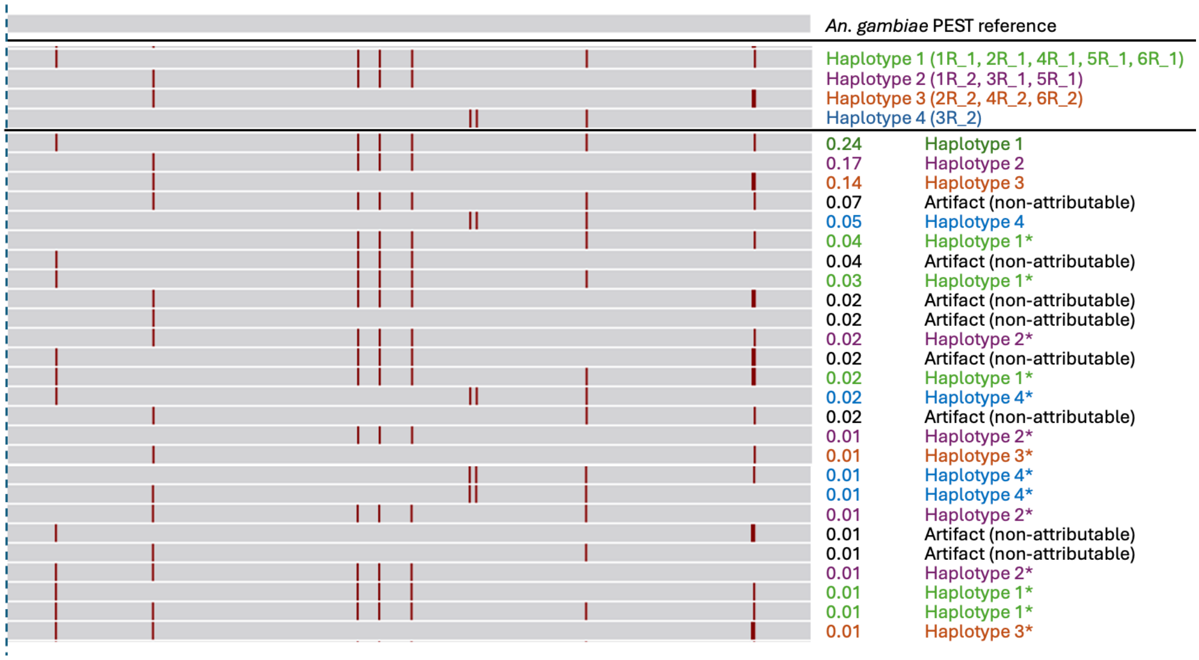

Supplementary Figure 3 - All alleles present at ≥0.5% of reads in pooled control samples on the right of the *zpg*-7280 cut site, with their relative abundances to each other. Alleles were aligned against the *Anopheles gambiae* PEST reference sequence, with the four expected haplotypes based on the sequences from individual samples shown underneath the PEST reference. The most common alleles match the first three expected haplotypes; the fifth most common allele matches Haplotype 4. The remaining alleles are either artifacts which have more than one mismatching base compared to the expected haplotypes, or have only 1 bp dissimilar to one of the expected haplotypes can therefore be reasonably assumed to originate from that haplotype (marked with an asterisk). This makes the pooled proportion of alleles: Haplotype 1 – 0.34, Haplotype 2 – 0.22, Haplotype 3 – 0.16, Haplotype 4 – 0.09.

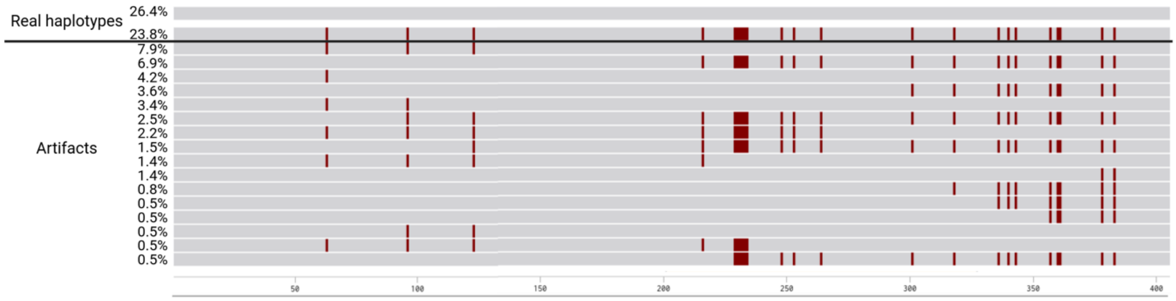

Supplementary Figure 4 – All alleles present at ≥0.5% relative abundance in control Sample 1, aligned against the most abundant allele in the sample. The alleles identified as artifacts map as chimeras of the two 'real' haplotypes in the sample (the two with the highest abundance).

Based on these controls, and on known parent sequences in the case of the *zpg*-7280/N'Gousso samples, we developed a strategy to classify reads as true haplotypes of the pooled offspring in the main study, detailed below.

Supplementary Note 2 – Determining true haplotypes in the main sample set

All reads present at above 0.5% relative abundance in the pools were aligned to the parent reads in *zpg*-7280/N'Gousso crosses. The most common read in *vas2*-5958 was assumed to be the donor, based on data from the *zpg*-7280 samples where this was always the case, and all reads above 0.5% relative abundance were aligned to it. For *vas2*-5958 pools the overall composition of the alleles was used to infer the likely alternate (recipient) chromosome, as allele pools are combinations of chimeric sequences of the true haplotypes. Relative abundances of alleles were recalculated by dividing the relative abundance of each allele with the sum of relative abundances of alleles over 0.5% for that sample. Alleles which had only one mismatched base from either the donor or recipient chromosome were grouped with their haplotype of origin. All alleles present at 0.5% relative abundance in each sample can be seen in **Figures S5-S10**.

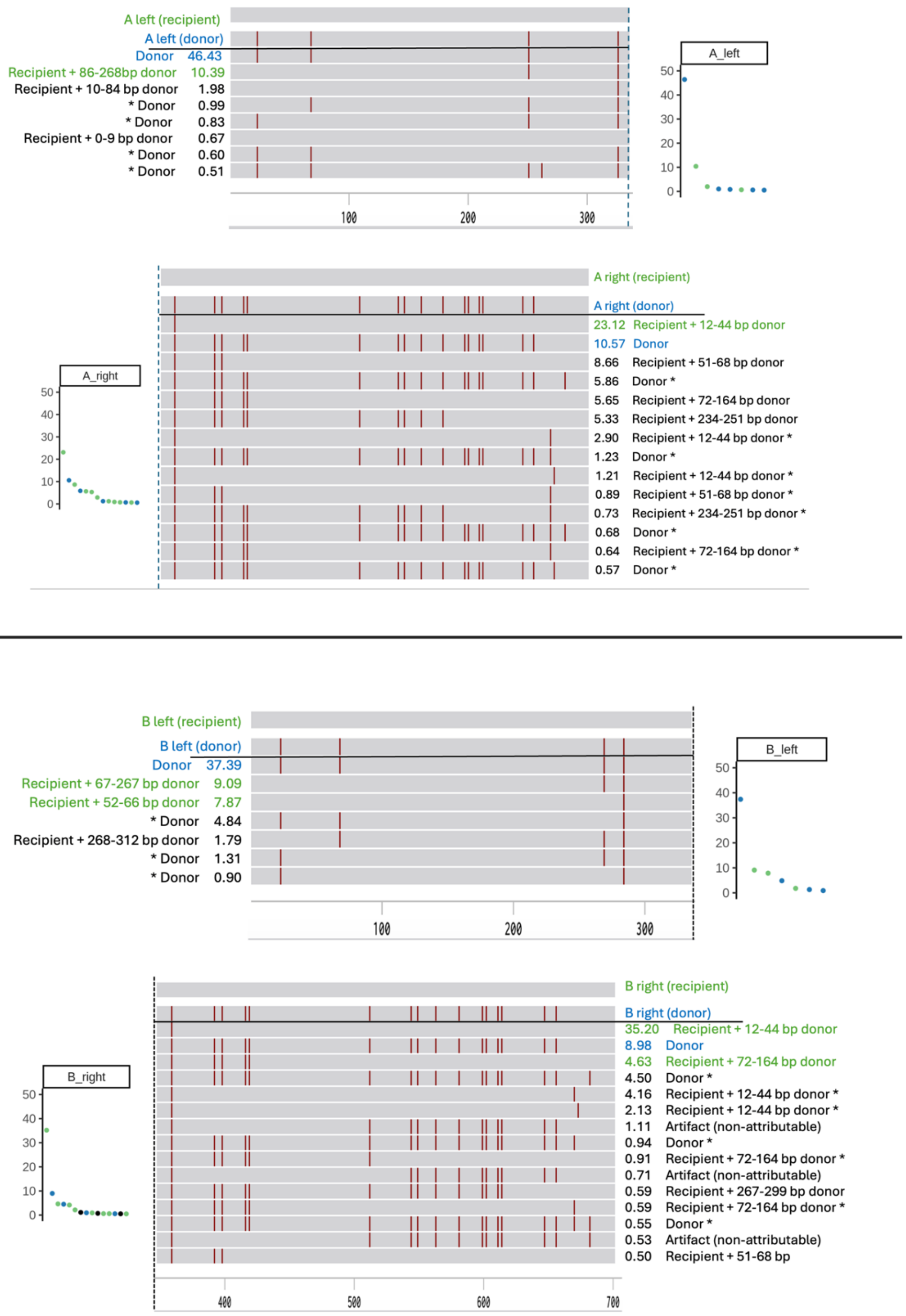

Supplementary Figure 5 – Candidate haplotypes in sample pools A and B (offspring of *zpg*-7280/N'Gousso females x G3 males). Alignments show the most abundant alleles aligned to the parent recipient (N'Gousso) chromosome; 'real' alleles are expected to match either the parent donor chromosome, parent recipient chromosome, or a mixture of the two, with higher abundance compared to other alleles. Numbers on the resection figures are the relative abundance percentage of each allele in the pool; dotted lines represent the cut site/beginning of the gene drive. Graphs show the abundance of each allele in the larval offspring pools, with identified donor and recipient alleles coloured blue and green respectively, and black indicating haplotypes not entirely matching an expected haplotype. Haplotypes marked with an asterisk were only one SNP different from an expected haplotype and were therefore labelled as (and grouped together with) that haplotype.

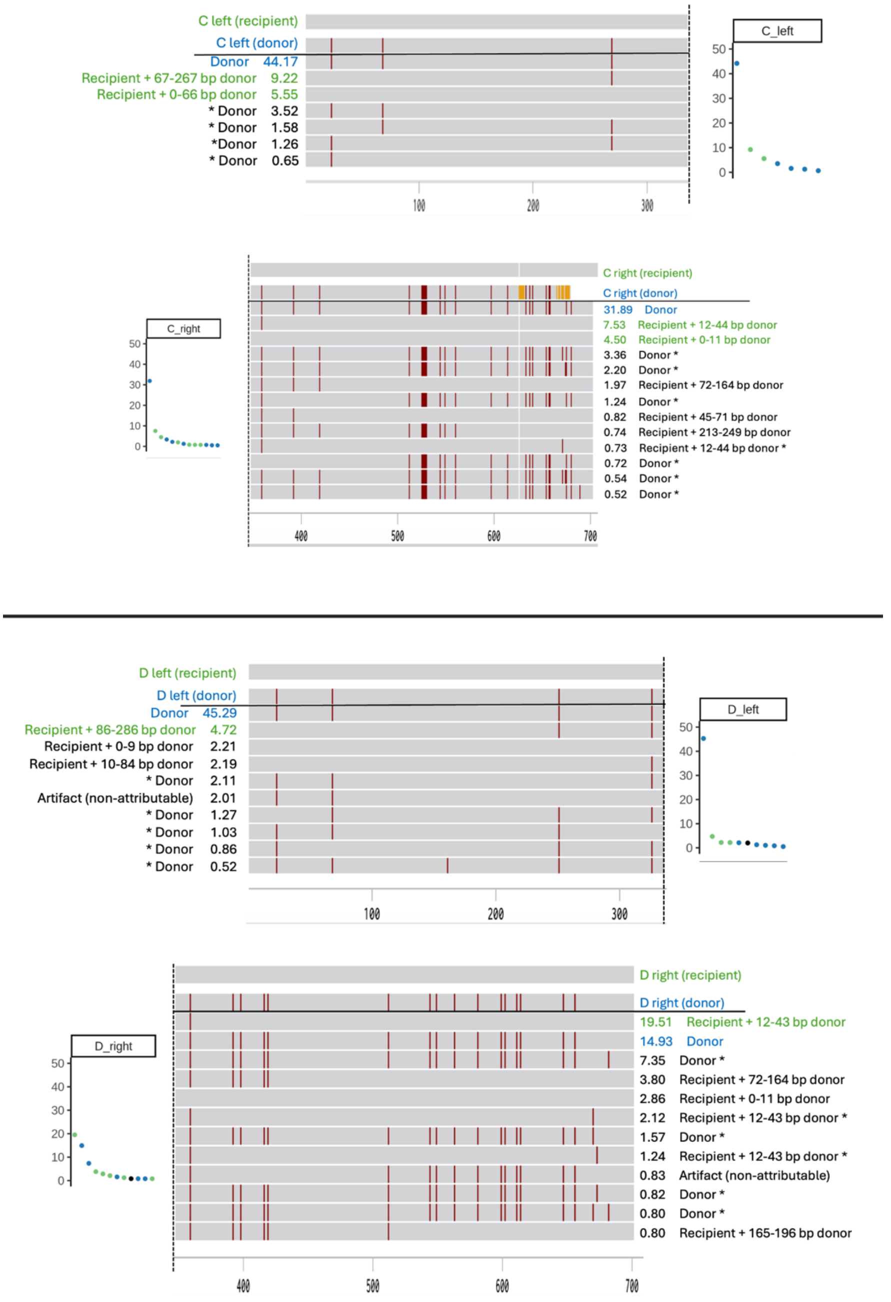

Supplementary Figure 6 - Candidate haplotypes in sample pools C and D (offspring of *zpg*-7280/N'Gousso females x G3 males). Alignments show the most abundant alleles aligned to the parent recipient (N'Gousso) chromosome; 'real' alleles are expected to match either the parent donor chromosome, parent recipient chromosome, or a mixture of the two, with higher abundance compared to other alleles. Numbers on the resection figures are the relative abundance percentage of each allele in the pool; dotted lines represent the cut site/beginning of the gene drive. Graphs show the abundance of each allele in the larval offspring pools, with identified donor and recipient alleles coloured blue and green respectively, and black indicating haplotypes not entirely matching an expected haplotype. Haplotypes marked with an asterisk were only one SNP different from an expected haplotype and were therefore labelled as (and grouped together with) that haplotype.

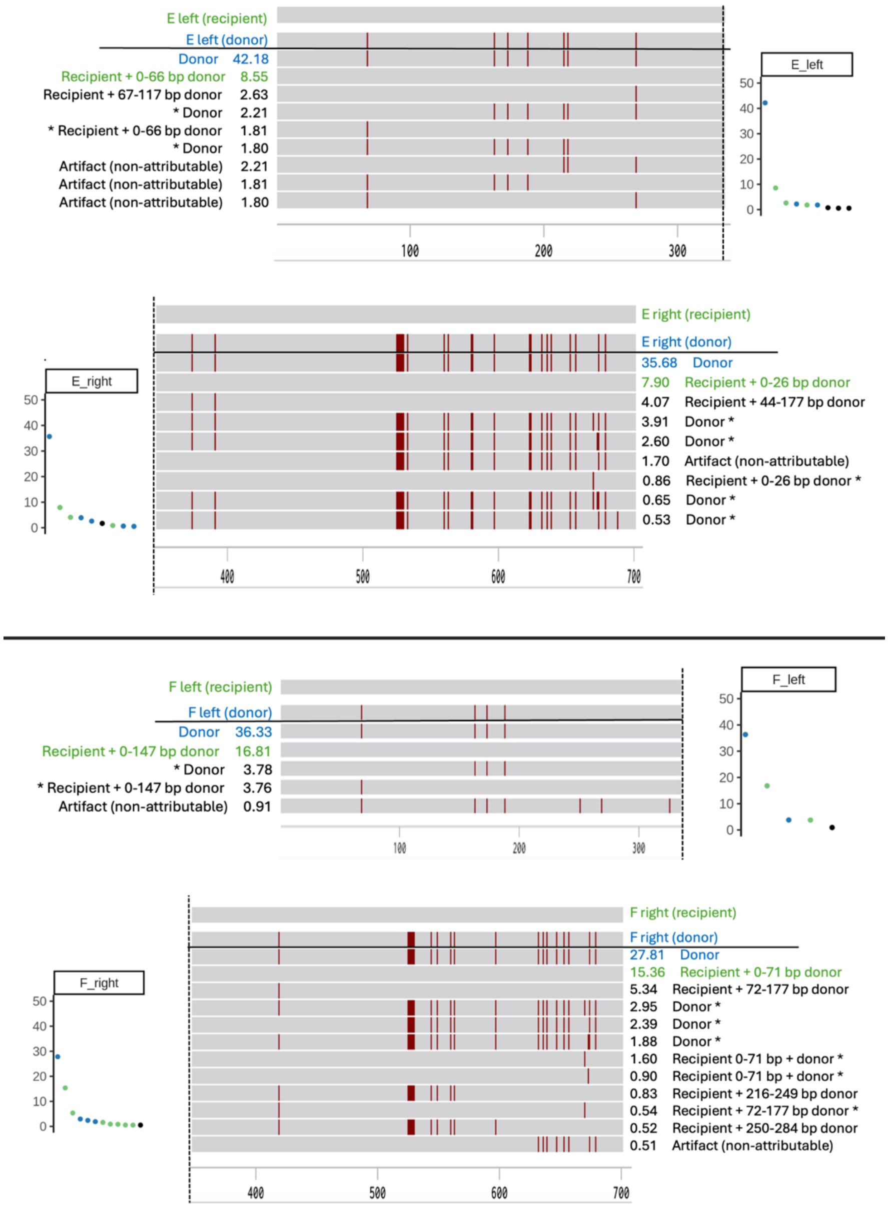

Supplementary Figure 7 - Candidate haplotypes in sample pools E and F (offspring of *zpg*-7280/N'Gousso females x G3 males). Alignments show the most abundant alleles aligned to the parent recipient (N'Gousso) chromosome; 'real' alleles are expected to match either the parent donor chromosome, parent recipient chromosome, or a mixture of the two, with higher abundance compared to other alleles. Numbers on the resection figures are the relative abundance percentage of each allele in the pool; dotted lines represent the cut site/beginning of the gene drive. Graphs show the abundance of each allele in the larval offspring pools, with identified donor and recipient alleles coloured blue and green respectively, and black indicating haplotypes not entirely matching an expected haplotype. Haplotypes marked with an asterisk were only one SNP different from an expected haplotype and were therefore labelled as (and grouped together with) that haplotype.

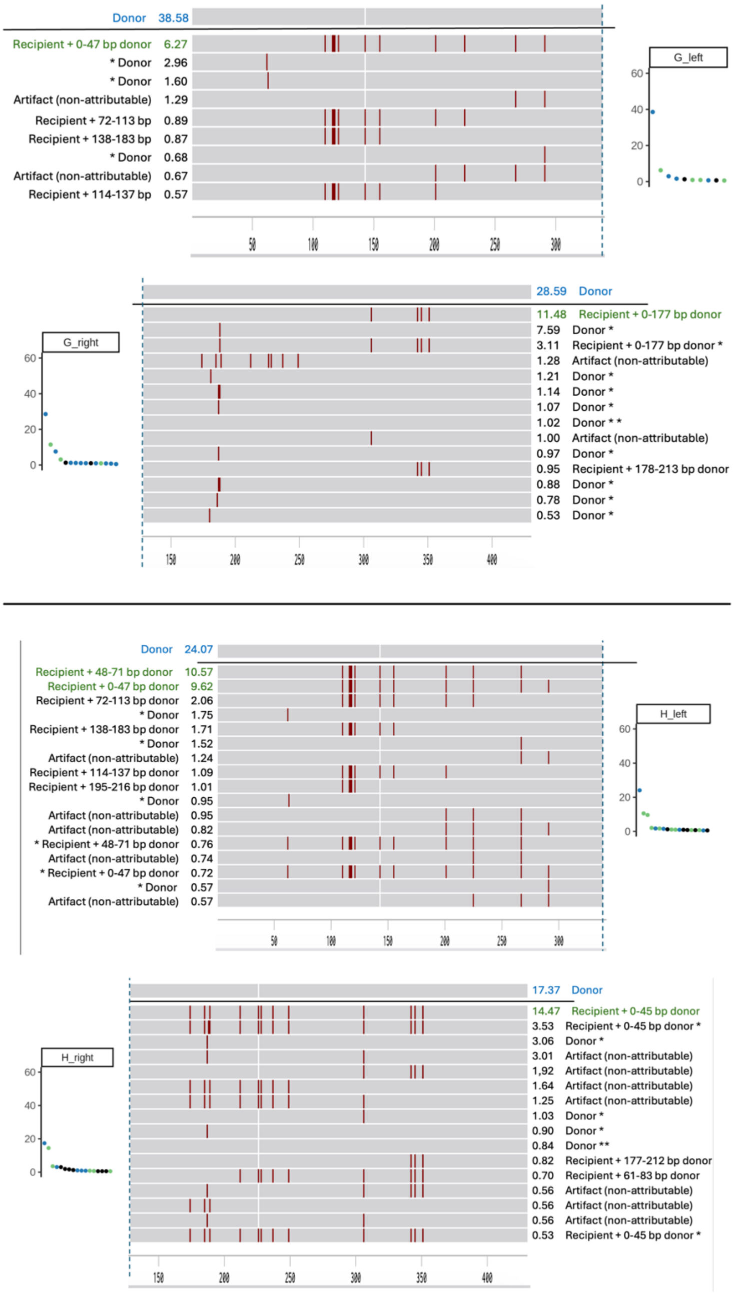

Supplementary Figure 8 - Candidate haplotypes in sample pools G and H (offspring of *vas2*-5958/N'Gousso males x G3 females). Alignments show the most abundant alleles aligned to the single most abundant allele, assumed to be the donor haplotype. 'Real' alleles are expected to be of a different haplotype to the most abundant allele, with potential variations involving the carryover of assumed 'donor' SNPs close to the cut site, with a higher abundance compared to the artifact alleles. Numbers on the resection figures are the relative abundance percentage of each allele in the pool; dotted lines represent the cut site/beginning of the gene drive. Graphs show the abundance of each allele in the larval offspring pools, with identified donor and recipient alleles coloured blue and green respectively, and black indicating haplotypes not entirely matching an expected haplotype. Haplotypes marked with an asterisk were only one SNP different from an expected haplotype and were therefore labelled as (and grouped together with) that haplotype.

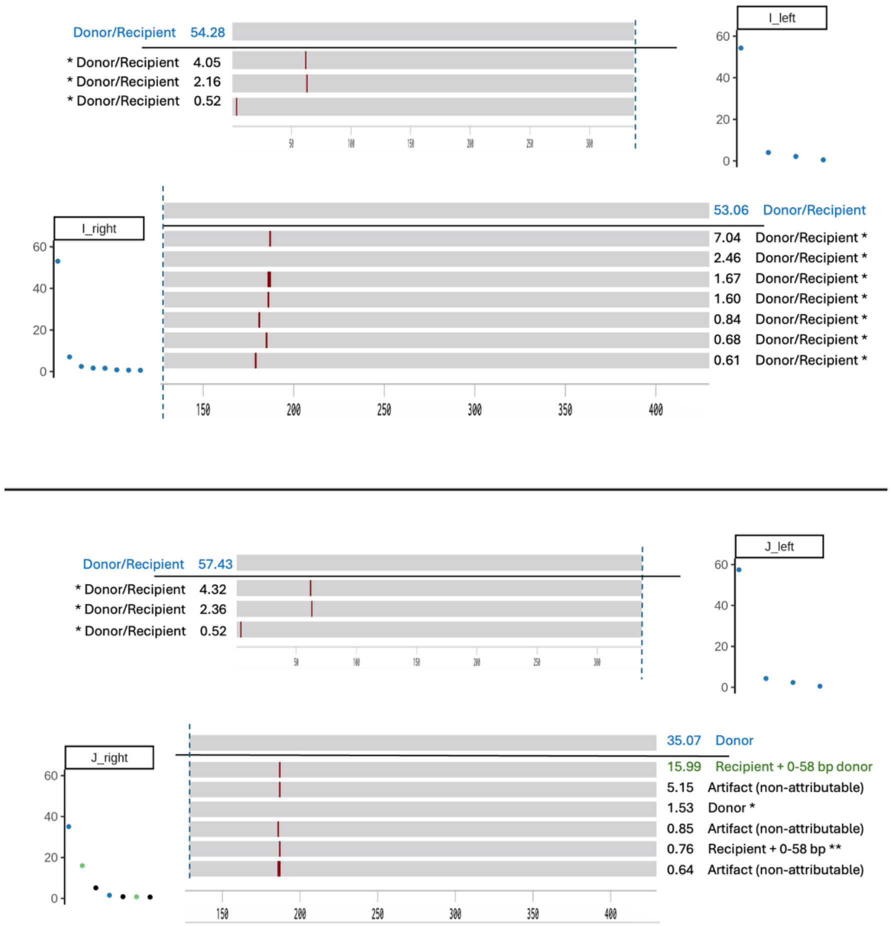

Supplementary Figure 9 - Candidate haplotypes in sample pools I and J (offspring of *vas2*-5958/N'Gousso males x G3 females). Alignments show the most abundant alleles aligned to the single most abundant allele, assumed to be the donor haplotype. Red lines indicate different nucleotides between the reference (donor) sequence and the other haplotypes; white lines indicate that the difference is an insertion, not a SNP. 'Real' alleles are expected to be of a different haplotype to the most abundant allele, with potential variations involving the carryover of assumed 'donor' SNPs close to the cut site, with a higher abundance compared to the artifact alleles. Numbers on the resection figures are the relative abundance percentage of each allele in the pool; dotted lines represent the cut site/beginning of the gene drive. Graphs show the abundance of each allele in the larval offspring pools, with identified donor and recipient alleles coloured blue and green respectively, and black indicating haplotypes not entirely matching an expected haplotype. Haplotypes marked with an asterisk were only one SNP different from an expected haplotype and were therefore labelled as (and grouped together with) that haplotype. In Pool I and the left of Pool J there was no distinct donor or recipient haplotype, likely due to a lack of SNPs between the parent chromosomes.

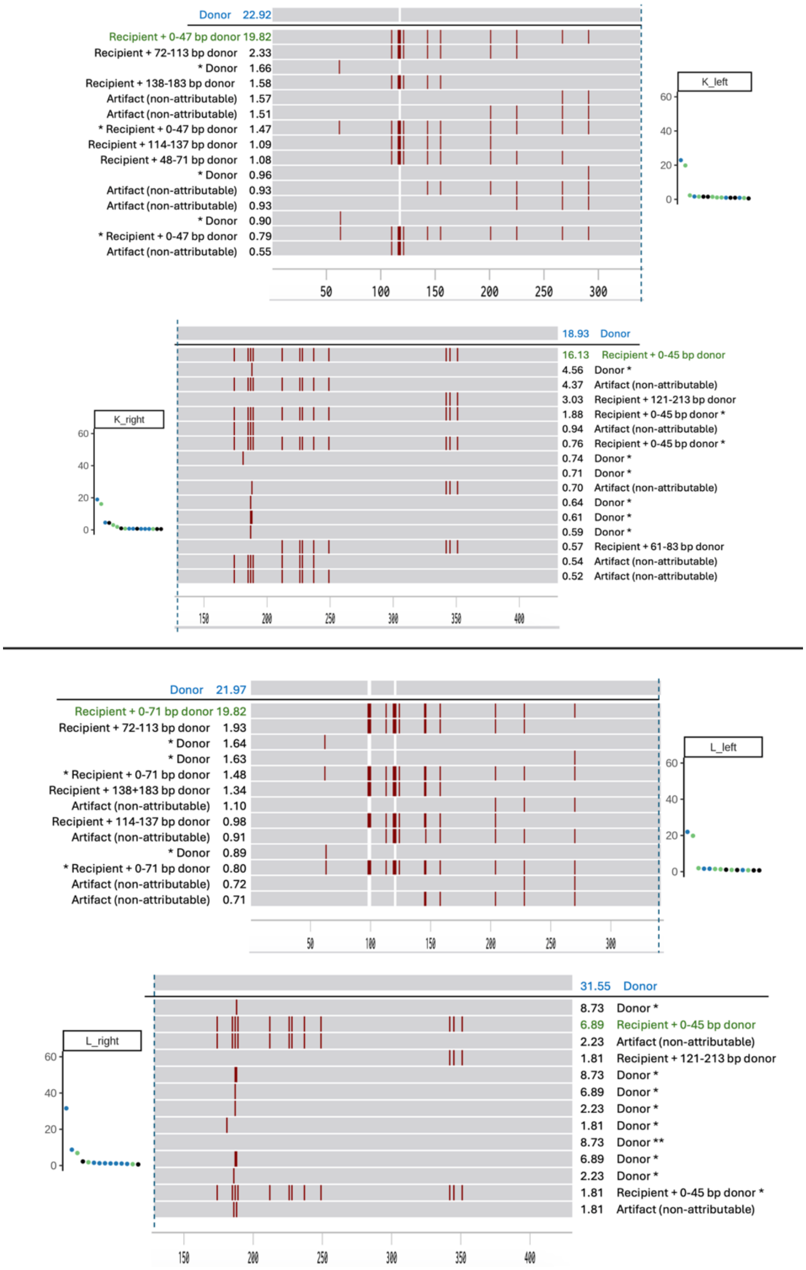

Supplementary Figure 10 - Candidate haplotypes in sample pools I and J (offspring of *vas2-5958*/N'Gousso males x G3 females). Alignments show the most abundant alleles aligned to the single most abundant allele, assumed to be the donor haplotype. Red lines indicate different nucleotides between the reference (donor) sequence and the other haplotypes; white lines indicate that the difference is an insertion, not a SNP. 'Real' alleles are expected to be of a different haplotype to the most abundant allele, with potential variations involving the carryover of assumed 'donor' SNPs close to the cut site, with a higher abundance compared to the artifact alleles. Numbers on the resection figures are the relative abundance percentage of each allele in the pool; dotted lines represent the cut site/beginning of the gene drive. Graphs show the abundance of each allele in the larval offspring pools, with identified donor and recipient alleles coloured blue and green respectively, and black indicating haplotypes not entirely matching an expected haplotype. Haplotypes marked with an asterisk were only one SNP different from an expected haplotype and were therefore labelled as (and grouped together with) that haplotype.

Supplementary Note 3 – Long read sequencing

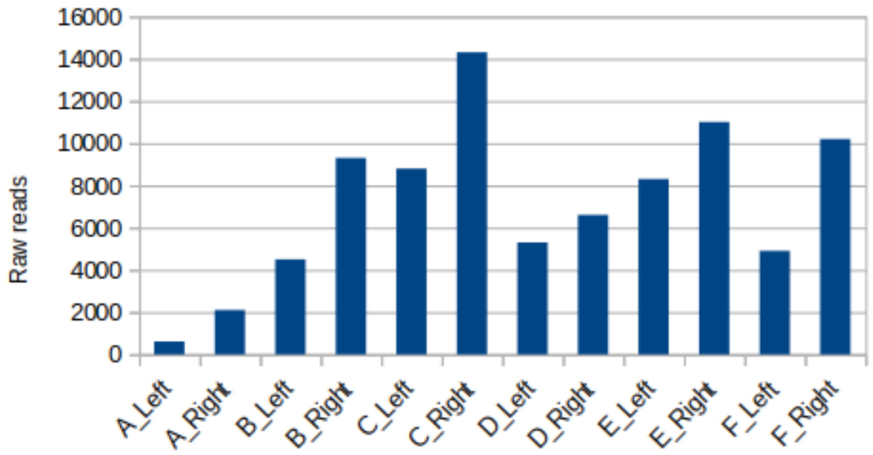

Supplementary Figure 11 - Raw read counts for long-read Nanopore MinION sequencing of 4 kb left and right of the gene drive in sample pools A-F.

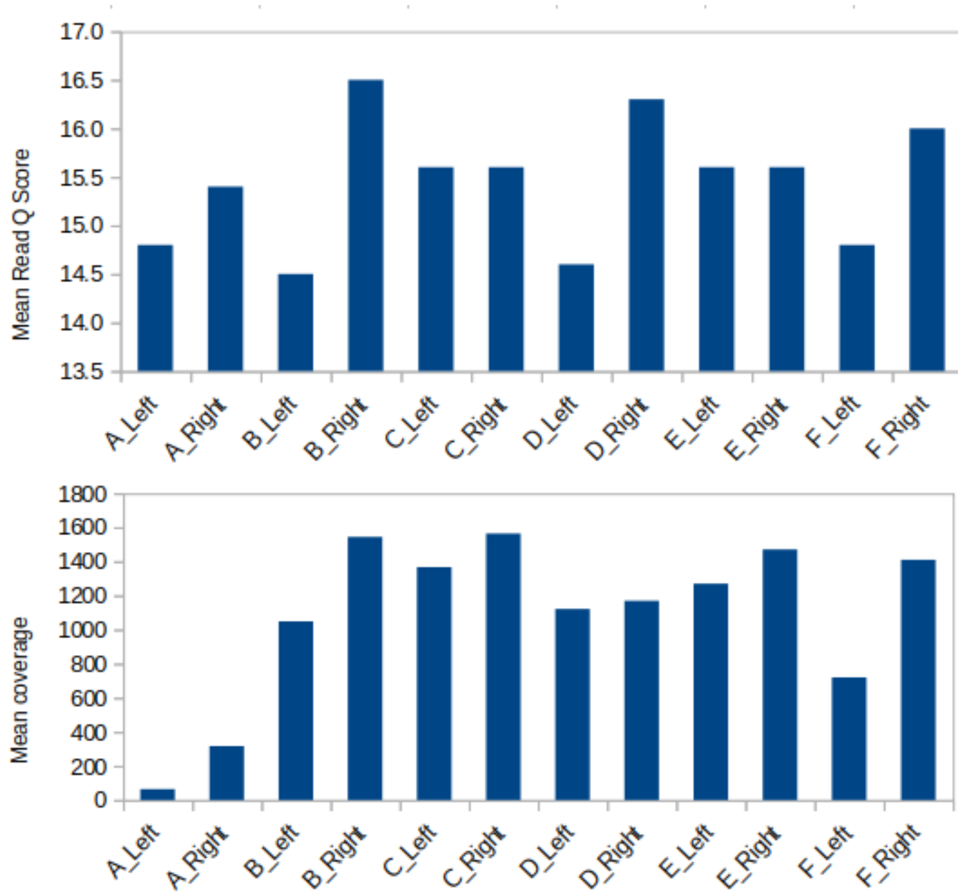

Supplementary Figure 12 - Mean read Q score (top) and mean coverage (bottom) for long-read Nanopore MinION sequencing of 4kb left and right of the gene drive in sample pools A-F.

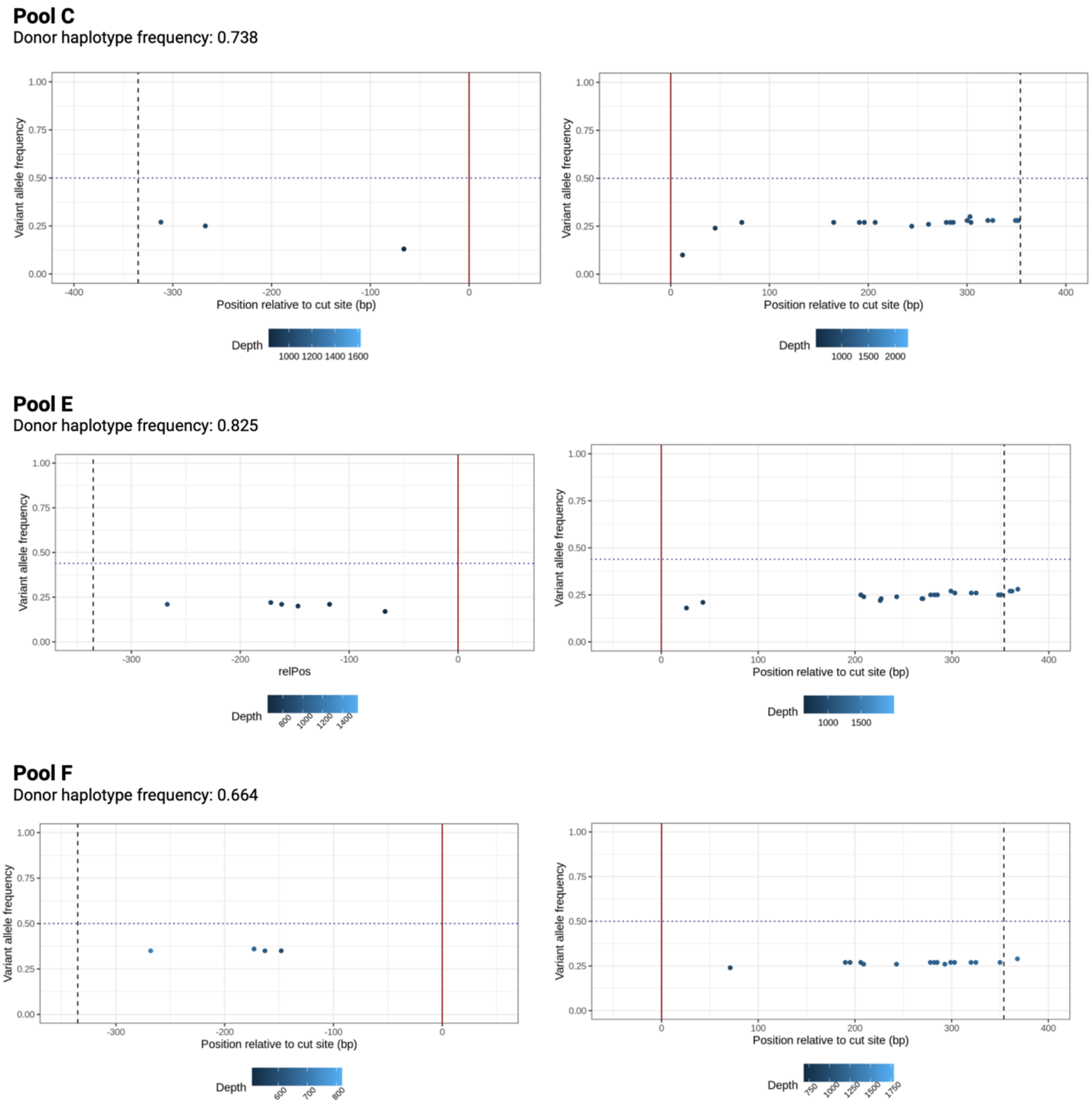

Supplementary Figure 13 - First ~350 bp next to the gene drive left and right of long read pools C, E and F. Red vertical line indicates the cut site/beginning of the gene drive, dashed vertical line indicates the end of the short read amplicon, and horizontal dotted line indicates the expected proportion of variants if homing was the only cause of inheritance bias and if there were no gene conversion tracts (GCTs) in the recipient chromosome. Pool C is the only one of these three pools with a GCT (one SNP at 12 bp to the right of the cut site and one at 67 bp to the left); this is visible in both graphs of Pool C as the closest point to the cut site in each, which both have a lower variant allele frequency than the rest of the variant sites.

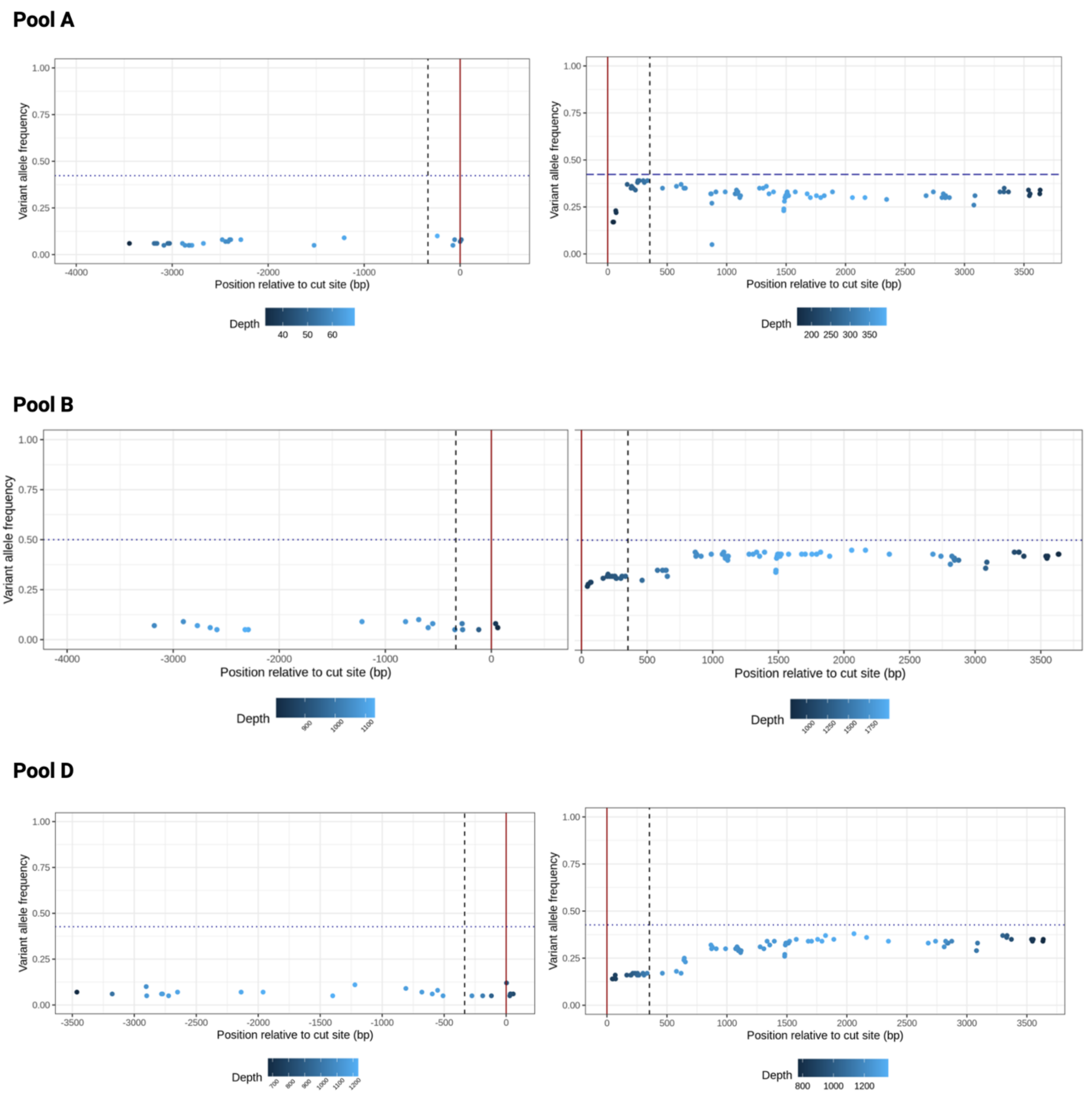

Supplementary Figure 14 – Variant allele frequency in pools A, B and D of zpg-7280/N'Gousso hybrid F2 offspring pools 4kb left and right of the gene drive insert site. These pools were removed from the main analysis due to a SNP in the short-read primer binding site precluding analysis of donor vs recipient chromosome proportions. Pool A on the left is analysed by comparing to the consensus sequence from the pool rather than the parent, due to low sequencing coverage near the cut site in the parent sequence. Pools B and D also required less stringent quality filtering (variants shown are >Q10), introducing erroneous SNPs within the gene drive sequence (beyond the red line).

The horizontal dotted line shows the expected proportion of variant alleles (which represent the recipient haplotypes) in each pool if all inheritance bias was due to gene drive homing rather than meiotic drive. The vertical dashed line shows the limit of the short read sequencing done before, with a vertical red line indicating the gene drive cut site.

Supplementary figures and tables

Supplementary Table 1 – Results of *zpg*-7280/N'Gousso and *vas2*-5958/N'Gousso hybrid backcrosses to G3, with information about the progeny pools and haplotypes. TLH = target locus heterology. Closest SNP position indicates the nucleotide position relative to the gene drive cut site of the closest SNP between the parent chromosomes, and therefore the limit of detection of small GCTs in each larval pool.

Pool	Gene drive inheritance (%)	# in pool	TLH in parent (%)	# of haplotypes	Larvae per haplotype	Closest SNP position	
						Left	Right
zpg-7280/N'Gousso x G3							
A	86.7	39	3.31	12	3.25	11	11
B	100.0	49	3.17	12	4.08	53	11
C	100.0	68	3.10	15	4.53	53	11
D	87.2	41	3.05	14	2.93	11	11
E	89.1	41	5.39	13	3.15	68	38
F	100.0	55	3.45	12	4.58	53	71
vas2-5958/N'Gousso x G3							
G	93.5	29	1.82	16	1.81	27	76
H	98.0	50	2.62	24	2.08	48	62
I	93.2	34	2.62	18	1.89	48	62
J	100.0	28	2.96	15	1.87	72	62

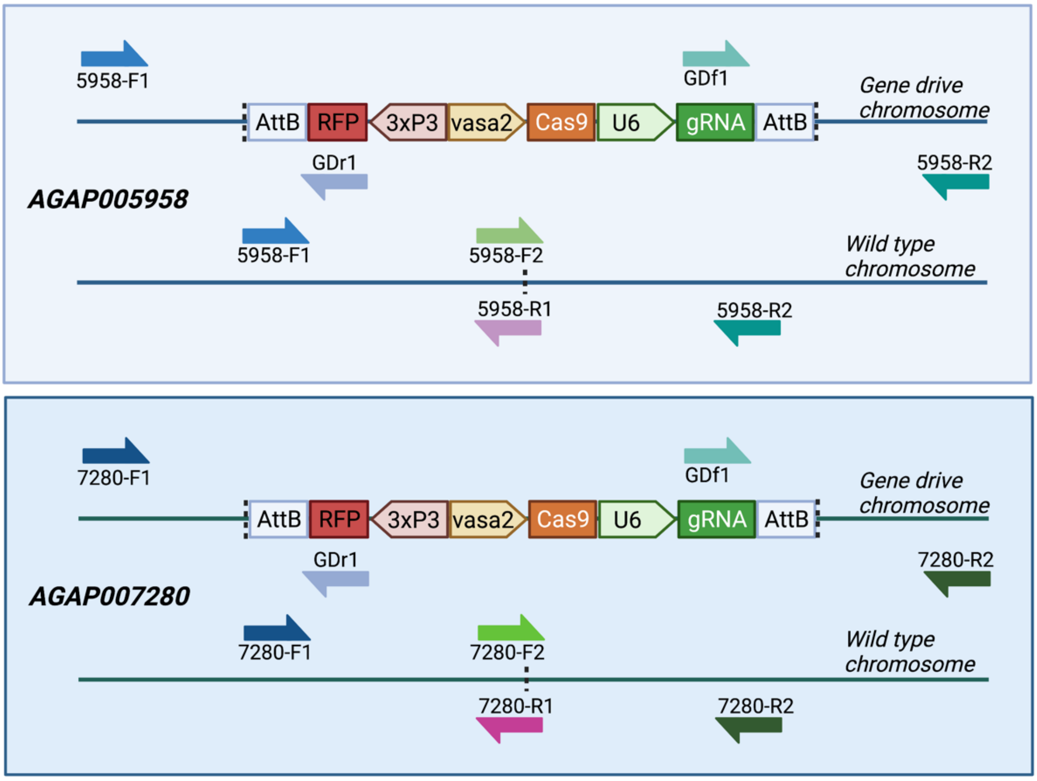

Supplementary Figure 15– Sequencing primer locations on both *vas2*-5958 and *zpg*-7280 gene drive and wild type target chromosomes.

Supplementary Table 2 - Primer sequences, with Illumina adapters in brackets. 4kb_left1 was used for F2 pools C and E, 4kb_left2 was used for F2 pool F and all *zpg*-7280 F1 samples after 4kb_left1 failed in a subset of samples due to an unknown SNP in the sequence.

**Name**	**Sequence (5'-3')**
5958-F1	(ACA CTC TTT CCC TAC ACG ACG CTC TTC CGA TCT) GCG CAC ATT AAG CCG TAC C
5958-R1	(GAC TGG AGT TCA GAC GTG TGC TCT TCC GAT CT) AGT GAC GAG ATA CTG GAG CC
5958-F2	(ACA CTC TTT CCC TAC ACG ACG CTC TTC CGA TCT) TCC TGG AGC AAC CGA TCA AG
5958-R2	(GAC TGG AGT TCA GAC GTG TGC TCT TCC GAT CT) TCG AGT AAA CCT TCT GGC CG
7280-F1	(ACA CTC TTT CCC TAC ACG ACG CTC TTC CGA TCT) GAC CGT TTG TGT GTC AGA GCA
7280-R1	(GAC TGG AGT TCA GAC GTG TGC TCT TCC GAT CT) GAA GCT CTC TGT GTG GCA CTA
7280-F2	(ACA CTC TTT CCC TAC ACG ACG CTC TTC CGA TCT) TGT GGG ATG GAT CAG ATG CT
7280-R2	(GAC TGG AGT TCA GAC GTG TGC TCT TCC GAT CT) CTC TGT ACT GAG GTC TGT TGT G
GDf1	(ACA CTC TTT CCC TAC ACG ACG CTC TTC CGA TCT) CAA CTT GAA AAA GTG GCA CCG
GDr1	(GAC TGG AGT TCA GAC GTG TGC TCT TCC GAT CT) CAA TGT ATC TTT CCG GAG CG
4kb_left1	GGT GAG CGA CTC GTA TGT CA
4kb_left2	CGA CTA CCG GGG TAT ACT GG
4kb_right	ATC TCA TCA CGC CTT CCG AC

Supplementary Table 3 – Annealing temperatures for all sequenced PCR products.

**Amplicon**	**Primers**	**Annealing temp (°C)**
vas2-5958 wild type (left)	5958-F1 + 5958-R1	63
vas2-5958 wild type (right)	5958-F2 + 5958-R2	64
vas2-5958 gene drive (left)	5958-F1 + GDr1	63
vas2-5958 gene drive (right)	GDf1 + 5958-R2	61
zpg-7280 wild type (left)	7280-F1 + 7280-R1	64
zpg-7280 wild type (right)	7280-F2 + 7280-R2	63
zpg-7280 gene drive (left)	7280-F1 + GDr1	63
zpg-7280 gene drive (right)	GDf1 + 7280-R2	61
zpg-7280 gene drive (4kb left_1)	4kb_left1 + GDr1	66
Zpg-7280 gene drive (4kb left_2)	4kb_left2 + GDr1	66
Zpg-7280 gene drive (4lb right)	GDf1 + 4kb_right	66

Supplementary Table 4 – Larval number and gene drive inheritance rate in each pool.

Amplicon pool	Gene drive locus	Number in pool	Gene drive inheritance rate
A	AGAP007280	39	86.7
B	AGAP007280	49	100.0
C	AGAP007280	68	100.0
D	AGAP007280	41	87.2
E	AGAP007280	41	89.1
F	AGAP007280	55	100.0
G	AGAP005958	29	93.5
H	AGAP005958	50	98.0
I	AGAP005958	34	100.0
J	AGAP005958	28	100.0
K	AGAP005958	82	93.2
L	AGAP005958	63	100.0
