# Supplementary material for: Homing gene drives can transfer rapidly between Anopheles gambiae strains with minimal carryover of flanking sequences
Source: Nat Commun. 2024 Aug 10;15:6846. doi: 10.1038/s41467-024-51225-9 (PMC11315913; doi:10.1038/s41467-024-51225-9)
Supplement: Supplementary file 2 — Reporting Summary [file 41467_2024_51225_MOESM2_ESM.pdf]

nature portfolio

Corresponding author(s): Tony Nolan
Last updated by author(s): Jul 21, 2024

Reporting Summary

Nature Portfolio wishes to improve the reproducibility of the work that we publish. This form provides structure for consistency and transparency in reporting. For further information on Nature Portfolio policies, see our Editorial Policies and the Editorial Policy Checklist.

Statistics

For all statistical analyses, confirm that the following items are present in the figure legend, table legend, main text, or Methods section.

n/a	Confirmed	
☐	☒	The exact sample size (*n*) for each experimental group/condition, given as a discrete number and unit of measurement
☒	☐	A statement on whether measurements were taken from distinct samples or whether the same sample was measured repeatedly
☐	☒	The statistical test(s) used AND whether they are one- or two-sided<br>*Only common tests should be described solely by name; describe more complex techniques in the Methods section.*
☒	☐	A description of all covariates tested
☐	☒	A description of any assumptions or corrections, such as tests of normality and adjustment for multiple comparisons
☐	☒	A full description of the statistical parameters including central tendency (e.g. means) or other basic estimates (e.g. regression coefficient) AND variation (e.g. standard deviation) or associated estimates of uncertainty (e.g. confidence intervals)
☐	☒	For null hypothesis testing, the test statistic (e.g. *F*, *t*, *r*) with confidence intervals, effect sizes, degrees of freedom and *P* value noted<br>*Give P values as exact values whenever suitable.*
☒	☐	For Bayesian analysis, information on the choice of priors and Markov chain Monte Carlo settings
☒	☐	For hierarchical and complex designs, identification of the appropriate level for tests and full reporting of outcomes
☒	☐	Estimates of effect sizes (e.g. Cohen's *d*, Pearson's *r*), indicating how they were calculated

Our web collection on statistics for biologists contains articles on many of the points above.

Software and code

Policy information about availability of computer code

Data collection	No software was used
Data analysis	CRISPResso2 (http://crispresso.pinellolab.org/) (v2.2.14) for allele generation from short read sequencing; EPI2ME wf-amplicons workflow (v. 1.0.2) (https://epi2me.nanoporetech.com) and bam-readcount (v. 1.0.1) (https://github.com/genome/bam-readcount) for variant calling in long-read data; Benchling (benchling.com) for aligning and visualising reads; Biorender (biorender.com) for producing figures; R (v. 4.1.2 for figures, v. 4.3.1 for statistics) (https://www.r-project.org) and ggplot2 (v. 3.3.6) for producing figures and statistical analysis.

For manuscripts utilizing custom algorithms or software that are central to the research but not yet described in published literature, software must be made available to editors and reviewers. We strongly encourage code deposition in a community repository (e.g. GitHub). See the Nature Portfolio guidelines for submitting code & software for further information.

Data

Policy information about availability of data

All manuscripts must include a data availability statement. This statement should provide the following information, where applicable:

- Accession codes, unique identifiers, or web links for publicly available datasets
- A description of any restrictions on data availability
- For clinical datasets or third party data, please ensure that the statement adheres to our policy

All raw sequencing data is publicly available in the NIH Sequence Read Archive under accession number PRJNA1043640 - https://www.ncbi.nlm.nih.gov/bioproject/PRJNA1043640.

Research involving human participants, their data, or biological material

Policy information about studies with human participants or human data. See also policy information about sex, gender (identity/presentation), and sexual orientation and race, ethnicity and racism.

Reporting on sex and gender	n/a
Reporting on race, ethnicity, or other socially relevant groupings	n/a
Population characteristics	n/a
Recruitment	n/a
Ethics oversight	n/a

Note that full information on the approval of the study protocol must also be provided in the manuscript.

Field-specific reporting

Please select the one below that is the best fit for your research. If you are not sure, read the appropriate sections before making your selection.

☒ Life sciences ☐ Behavioural & social sciences ☐ Ecological, evolutionary & environmental sciences

For a reference copy of the document with all sections, see nature.com/documents/nr-reporting-summary-flat.pdf

Life sciences study design

All studies must disclose on these points even when the disclosure is negative.

Sample size	Sample sizes were chosen based on a combination of the number of pools necessary to see a range of homing events, and cost effectiveness for sequencing of parents and pools from two different gene drive lines.
Data exclusions	No data was excluded other than that which has been fully described in the paper.
Replication	The six pools were biological replicates, standardised by being from the same generation of the line, and results would be reproducible using the same mosquito strains.
Randomization	Pools were partially randomised by randomly choosing females from group cages to put into single depositions. Choice of which pools to take forward for sequencing was not entirely random; they were chosen to give a range of gene drive inheritance rates (from 87-100%) in order to control for this variable in the results.
Blinding	No blinding was required for this study as there was no way of introducing bias based on non-blinding.

Reporting for specific materials, systems and methods

We require information from authors about some types of materials, experimental systems and methods used in many studies. Here, indicate whether each material, system or method listed is relevant to your study. If you are not sure if a list item applies to your research, read the appropriate section before selecting a response.

Materials & experimental systems

n/a	Involved in the study
☒	☐ Antibodies
☒	☐ Eukaryotic cell lines
☒	☐ Palaeontology and archaeology
☐	☒ Animals and other organisms
☒	☐ Clinical data
☒	☐ Dual use research of concern
☒	☐ Plants

Methods

n/a	Involved in the study
☒	☐ ChIP-seq
☒	☐ Flow cytometry
☒	☐ MRI-based neuroimaging

Animals and other research organisms

Policy information about studies involving animals; ARRIVE guidelines recommended for reporting animal research, and Sex and Gender in Research

Laboratory animals	Mosquitoes (insects) of the Anopheles gambiae species were used.
Wild animals	no wild animals were used
Reporting on sex	Both sexes were analysed in different experiments and the direction of genetic crosses are described explicitly in the manuscript.
Field-collected samples	No field collected samples were used in this study but the nature and origin of laboratory colonies of mosquitoes is described in the methods.
Ethics oversight	Research involving vertebrate animals and some non-vertebrate animals is covered by the Animals (Scientific Procedures) Act 1986 and must be performed under a license from the Home Office. No such animals were used in this study. Invertebrate animals such as the fruit fly, worms and mosquitoes, which are all commonly used in biomedical research, are not protected by the law.

Note that full information on the approval of the study protocol must also be provided in the manuscript.

Plants

Seed stocks	NA
Novel plant genotypes	NA
Authentication	NA
