# Supplementary material for: Homing gene drives can transfer rapidly between Anopheles gambiae strains with minimal carryover of flanking sequences
Source: Nat Commun. 2024 Aug 10;15:6846. doi: 10.1038/s41467-024-51225-9 (PMC11315913; doi:10.1038/s41467-024-51225-9)
Supplement: Supplementary file 4 — Description of Additional Supplementary Files [file 41467_2024_51225_MOESM4_ESM.pdf]

<u>Description of Additional Supplementary Files</u>

Supplementary Data 1 – **All haplotype sequences present at >0.5% relative abundance.** Table S5 is submitted separately as an Excel spreadsheet, with the following columns:
Sample – The pool letter (A-L) and whether the sequence is from the left or right of the gene drive cut site.
Haplotype – The identifier for each individual sequence, numbered from most to least abundant.
Gene drive – The gene drive contained by the pool (either *zpg*-7280 or *vas2*-5958).
% of total reads – The percentage of the total reads made up of each unique sequence (before removal of any reads present under 0.5%).
Sequence – The nucleotide sequence
Classification – Whether the sequence matched the gene drive Donor chromosome, the Recipient chromosome, or was classified as a Minor allele (making up <10% of the filtered reads)
Classification2 – How the sequence was classified after ignoring any differences of a single nucleotide from an identified true haplotype in the pool, which were likely the result of PCR error. Any sequences with >1 nucleotide different to an identified true haplotype were classed as Artifacts.
